# Effects of Acute Beetroot Juice Supplementation and Exercise on Cardiovascular Function in Healthy Men in Preliminary Study: A Randomized, Double-Blinded, Placebo-Controlled, and Crossover Trial

**DOI:** 10.3390/healthcare12131240

**Published:** 2024-06-21

**Authors:** Xie Yuschen, Jae-Ho Choi, Jisoo Seo, Yerin Sun, Eunjoo Lee, Sung-Woo Kim, Hun-Young Park

**Affiliations:** 1 Department of Sports Medicine and Science, Graduate School, Konkuk University, 120 Neungdong-ro, Gwangjin-gu, Seoul 05029, Republic of Korea; xieyuchen@konkuk.ac.kr (X.Y.); zas1135@konkuk.ac.kr (J.-H.C.); zxsd23@konkuk.ac.kr (J.S.); edre82@konkuk.ac.kr (Y.S.); eunjooo@konkuk.ac.kr (E.L.); kswrha@konkuk.ac.kr (S.-W.K.); 2 Physical Activity and Performance Institute, Konkuk University, 120 Neungdong-ro, Gwangjin-gu, Seoul 05029, Republic of Korea

**Keywords:** beetroot juice, nitrate, exercise, cardiovascular function, pulse wave velocity, flow mediate dilation, healthy men

## Abstract

Nitrate-rich beetroot juice (NRBRJ) can potentially enhance exercise performance and improve cardiovascular function, leading to an increased use of NRBRJ over the years. However, the combined effects of NRBRJ supplementation and exercise on cardiovascular function remain unclear. Therefore, this study compared cardiovascular function responses to submaximal exercise with either placebo (PLA) or NRBRJ supplementation in healthy men. Twelve healthy men (aged 25.2 ± 2.3 years) completed the 30-min submaximal cycle ergometer exercise trials corresponding to 70% maximal heart rate (HRmax) with either PLA or NRBRJ supplementation in a random order. The mean exercise load, heart rate (HR), stroke volume (SV), cardiac output (CO), systolic blood pressure (SBP), diastolic blood pressure (DBP), mean arterial pressure (MAP), and total peripheral resistance (TPR) were measured during exercise. The brachial–ankle pulse wave velocity (baPWV) and flow-mediated dilation (FMD) were measured before and after exercise. NRBRJ supplementation was more effective than PLA in increasing the mean exercise load and decreasing DBP and MAP during submaximal exercise. Furthermore, baPWV decreased in the NRBRJ trial and was considerably lower after exercise in the NRBRJ-supplemented group than in the PLA-supplemented group. FMD significantly increased in the PLA and NRBRJ trials; however, NRBRJ supplementation demonstrated a significantly higher FMD before and after exercise than PLA supplementation. In conclusion, acute NRBRJ supplementation and exercise were more effective than PLA supplementation and exercise in improving aerobic exercise capacity and cardiovascular function in healthy men.

## 1. Introduction

Beetroot contains a higher amount of nitrate ($NO_{3}^{-}$) than other vegetables such as lettuce, spinach, endive, and turnip [1,2,3]. $NO_{3}^{-}$ intake can improve athletic performance, and over the years, the use of nitrate-rich beetroot juice (NRBRJ) from *Beta vulgris* by athletes in endurance training has increased [4]. After NRBRJ intake, $NO_{3}^{-}$ in the body circulates in the plasma, is concentrated by approximately 25% in the salivary glands, and is secreted into the mouth, of which 20% is converted from $NO_{3}^{-}$ to nitrite by symbiotic bacteria on the tongue and swallowed [5]. The converted nitrite is directly absorbed from the stomach or is partially reduced to nitric oxide (NO) by stomach acid [5]. The NO and nitrite produced are absorbed into the blood vessels through systemic circulation, dilating blood vessels and improving cardiovascular functions such as reducing blood pressure (BP) [6]. Additionally, it was found to destroy reactive nitrogen (RNS) and oxygen (ROS) species, suggesting that absorbed $NO_{3}^{-}$ from NRBRJ consumption also provides an antioxidant effect [7,8,9].

NO can activate cardiomyocytes (e.g., decreased oxygen consumption and decreased apoptosis), coronary circulation (e.g., vasodilatation, increase in coronary blood flow, antithrombotic and anti-inflammatory action, and endothelial dysfunction improvement), myocardium, and the conductive system (e.g., negative inotropic and chronotropic effects) within the human body [10]. These effects are mediated through the NO/cGMP pathway, local mitochondrial metabolism regulation, calcium ion regulation in myocardial cells, and autonomic nervous system control [10].

Previous studies have demonstrated that NRBRJ supplementation enhances vascular function through various mechanisms (inhibit platelet aggregation, promotion of vasodilation, inhibition of vasoconstriction, and production of free radicals) [11,12,13]. In some studies, the largest BP reduction occurred 2–3 h after NRBRJ ingestion, equivalent to the peak plasma nitrite ($NO_{2}^{-}$) concentration observed [14,15,16]. As such, $NO_{2}^{-}$ may provide vasculoprotective effects when classical NO synthesis is impaired [14]. This acutely lowers arterial BP, supplements endothelial function (measured using flow-mediated dilation, FMD) during ischemia, and inhibits platelet aggregation due to NO bioconversion [14].

Regular exercise is known to reduce the risk of cardiovascular disease by improving vascular function by regulating oxidative stress (OS) and endothelial function [17,18]. One of the mechanisms by which exercise improves vascular endothelial function is an increase in blood flow and shear stress, which may improve vascular homeostasis through the increase in NO bioavailability in the endothelium [19,20]. Furthermore, there is an improvement in the ability to produce vasodilators such as NO and prostacyclin, which may influence endothelial function [21]. Previous studies conducted through this mechanism reported that regular exercise decreased total peripheral resistance (TPR), heart rate (HR), and BP and increased arterial baroreflex sensitivity and cardiovascular variability [12].

Several studies have been conducted on the effects of NRBRJ supplementation and exercise on athletic performance, and the rationale is based on several factors. First, after NRBRJ supplementation, NO and $NO_{2}^{-}$ increase blood flow [22,23,24], which can increase oxygenation to the muscles, thereby improving exercise capacity [25,26]. Second, the plasma $NO_{2}^{-}$ bioavailability is increased, which acts as a regulator of hypoxic signaling for NO-induced vasodilatory capacity [7,24,27]. Third, the hypoxic and acidic conditions noted during exercise promote $NO_{2}^{-}$-to-NO reduction and enhance the physiological effects of exogenously ingested NRBRJ [23]. These effects can improve exercise capacity by altering the metabolic and hemodynamic responses of muscles to exercise [28,29]. Previous studies have comprehensively validated the effects of NRBRJ supplementation and exercise on exercise capacity. however, there is a paucity of relevant studies on changes in cardiovascular function before, during, and after exercise [30]. In particular, while there are many studies related to blood pressure, there is a lack of research on changes in arterial stiffness and endothelial function before and after exercise following NRBRJ supplementation [31]. This study aims to fill this research gap and investigate the additional effects of NRBRJ supplementation and exercise on cardiovascular function in healthy men.

Therefore, this study aimed to examine the additive effects of NRBRJ supplementation and exercise on cardiovascular function in healthy men compared to effects from placebo (PLA) supplementation and exercise. The hypothesis of the present study was that NRBRJ supplementation and exercise may enhance cardiovascular function in healthy men compared with PLA supplementation and exercise.

## 2. Materials and Methods

### 2.1. Participants

The participants included twelve healthy men who were nonsmokers and non-obese; had no history of musculoskeletal, cardiovascular, or pulmonary diseases; did not participate in any planned exercise program; and had not consumed any dietary supplements 6 months prior. All healthy men were identified as healthy via the Physical Activity Readiness Questionnaire for Everyone and were restricted from intake of cabbages (i.e., broccoli, cauliflower, red cabbage, etc.) for 2 weeks before study participation [16]. However, the physical activity and dietary intake of all participants were not investigated during the study period. The participants were briefed on the purpose and process of the study, and informed consent was obtained before initiation of the study.

In this study, the sample size was determined with the goal of achieving a test power exceeding 80%. A previous study involving healthy adults reported a mean change in blood pressure between the $NO_{3}^{-}$ group and the PLA group as 10 mmHg, with a standard deviation (SD) of 9 mmHg [32]. Based on these findings, a G-power test was conducted with a significance level (α) of 0.05, a power level (1 − β) set to 0.8, and an effect size (dz) set to 1.11. The calculated sample size required to meet these criteria was determined to be a total of 10 participants. However, we considered dropouts and selected 12 research participants. The expected test power at this sample size exceeds 80%, and the normality test further supports the reasonableness of the selected sample size.

The physical characteristics of the participants are illustrated in Table 1. The trial was registered and disclosed to the Institutional Review Board of Konkuk University (7001355-202301-E-616) in Korea and was conducted according to the Declaration of Helsinki guidelines.

### 2.2. Study Design

**Figure 1:**
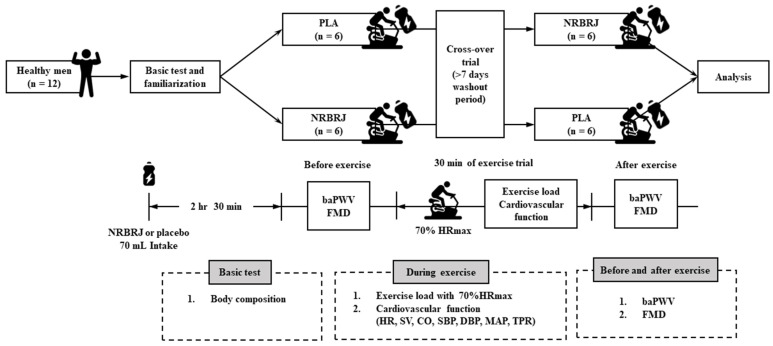
Study design. NRBRJ: nitrate-rich beetroot juice; PLA: placebo; HRmax: maximal heart rate; HR: heart rate; SV: stroke volume; CO: cardiac output; SBP: systolic blood pressure; DBP: diastolic blood pressure; MAP: mean arterial pressure; TPR: total peripheral resistance; baPWV: brachial–ankle pulse wave velocity; FMD: flow-mediated dilation.

The study design is shown in Figure 1. During the experimental period, all participants visited the laboratory thrice.

During the first visit, the participants fasted for >4 h, and their height and body composition were measured the morning after stabilization. Subsequently, all of the participants underwent a familiarization trial under non-intake conditions using the exercise protocol before the main trial for exercise program adaptation. The exercise protocol consisted of 30 min of endurance exercise on a cycle ergometer (Aero bike 75XLII; Konami Corporation, Tokyo, Japan) at the same HR level equivalent to 70% maximal HR (70% HRmax), calculated using the Miyashita formula (male = 209 − 0.69 × age) [33]. The target HR of the participants, 70% HRmax, was 136.5 ± 1.5 bpm.

In the study, we searched several studies for study design and found that exercising on a treadmill has limitations in measuring blood pressure. Therefore, a cycle ergometer was selected to obtain accurate data for measuring both blood pressure and cardiac function. The chosen exercise type and duration (30 min) were similar, and 70% of HRmax was used, referring to previous studies that evaluated cardiac function during exercise [33]. In addition, according to the American College of Sports Medicine (ACSM) guidelines, 70% of HRmax is considered a reasonable exercise intensity as it essentially corresponds to the medium-intensity level recommended for improving health [34].

**Figure 2:**
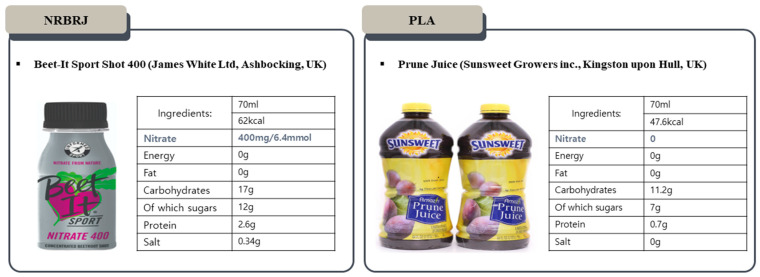
Details of the PLA and NRBRJ supplement. NRBRJ: nitrate-rich beetroot juice; PLA: placebo.

On the second and third visits, all participants were instructed to fast and prohibit mouthwashing (gargle) and brushing for 12 h prior to testing in the laboratory [16]. Moreover, they were instructed to refrain from physical activity for 24 h prior to the test. All participants were asked to consume PLA and/or NRBRJ 2.5 h before exercise. Figure 2 illustrates the details of the PLA and NRBRJ supplements [35]. The NRBRJ supplement was 70 mL of Beet-It Sport Shot 400 (James White Ltd., Ashbocking, UK), which contains 400 mg of $NO_{3}^{-}$ [35]. Prune juice (Sunsweet Growers Inc., Kingston upon Hull, UK) was selected as a PLA supplement because of its negligible $NO_{3}^{-}$ levels (<0.01 mM) and similar consistency and color to those of NRBRJ supplementation [36]. Additionally, prior research has indicated that the carbohydrate and fiber content of the two supplements does not significantly affect the experiment [36]. Due to the red color of beetroot, side effects of NCBRJ consumption include red urine and red stool [35]. After 2.5 h of PLA and/or NRBRJ supplementation, all participants were evaluated for brachial–ankle pulse wave velocity (baPWV) and FMD before and after 30 min of endurance exercise. Moreover, the participants performed endurance exercises in a crossover randomized order with PLA and/or NRBRJ supplementation. A 7-day washout period was implemented between the second and third visits to ensure that the effects of the ingested substances did not carry over [15]. During 30 min of the endurance exercise session, the following parameters were measured: mean exercise load, TPR, cardiac output (CO), stroke volume (SV), HR, systolic blood pressure (SBP), diastolic blood pressure (DBP), and mean arterial pressure (MAP). All exercise parameters were measured at rest and at 5, 10, 20, and 30 min during the endurance exercise.

In all trials, the endurance exercise was conducted at the same temperature and humidity (23 ± 1 °C, 50% ± 5%) (environmental control chamber; NCTC-1, Nara Control, Seoul, Republic of Korea).

### 2.3. Outcome Measurements

#### 2.3.1. Height and Body Composition

Height and body composition were measured using the Inbody 770 (Inbody, Seoul, Republic of Korea) in a light top and bottom. After fasting for at least 4 h before the test, the participants visited the laboratory. Each participant removed any metallic material adhering to their body, stood on the measuring device, and stepped on the electrode with bare feet. Height was obtained with the participants holding the handle of the measuring device with both hands and in an upright position with arms and legs slightly apart. Height (cm), body weight (kg), body mass index (kg/m^2^), fat-free mass(kg), body fat mass (kg), and body fat percentage (%) were measured.

#### 2.3.2. Mean Exercise Load during Exercise

The mean exercise load corresponding to 70% HRmax during 30 min of endurance exercise on a cycle ergometer was automatically calculated using the instrument.

#### 2.3.3. Cardiovascular Function during Exercise

A thoracic bioelectrical impedance device (PhysioFlow PF-05, Paris, France) was used to noninvasively measure HR and SV, which are cardiovascular function parameters. The bioimpedance device consisted of two impedance cardiography electrodes placed above the supraclavicular fossa at the base of the left side of the neck, two electrocardiography electrodes used to record an electrocardiogram (ECG), and two electrodes placed at the xiphoid process. Impedance cardiography measures changes in thoracic impedance during the cardiac cycle to calculate SV [37]. PhysioFlow is an impedance technique that emits high-frequency (75 kHz) and low-magnitude (1.8 mA) alternating electrical currents via skin electrodes during the cardiac cycle. This resulted in a waveform from which SV was calculated [38]. The HR is obtained from the R-R interval determined from the first derivative of the ECG and CO as calculated according to the following formula: CO = HR × SV. At rest and during exercise, PhysioFlow PF-05 was validated against the direct Fick method [38]. The brachial artery BP was measured via a manual sphygmomanometer UM-102 (Boryung A&D Systems, Seoul, Republic of Korea). The BP of each participant was measured by the same investigator throughout the experiment. MAP and TPR were calculated using the formula MAP = [(SBP − DBP) × 1/3]/DBP, TPR = MAP/CO. HR, SV, CO, SBP, DBP, MAP, and TPR were analyzed at rest and at 5, 10, 20, and 30 min during endurance exercise on a cycle ergometer.

The PWV is the best parameter for evaluating arterial stiffness. Therefore, the baPWV was measured using an automatic Oscillo metric device (VP-1000plus, Omron, Osaka, Japan) before and after exercise. Before measurement, participants wore sensor cuffs on both the upper arms and ankles. For HR monitoring, phonocardiogram electrodes were worn on the left edge of the sternum and ECG electrodes on both wrists. Resting baPWV was measured after resting for at least 30 min, and after-exercise baPWV was measured within 10 min of the completion of exercise for each trial [39].

FMD refers to the dilation (widening) of an artery when the blood flow increases in that artery. The release of NO by endothelial cells is the primary cause of FMD. Therefore, FMD of the brachial artery was measured to evaluate vascular endothelial function using noninvasive Doppler ultrasound (UNEX-EF, Tokyo, Japan) before and after exercise. After the measurement, blood was removed for 5 min by increasing the blood pressure by 50 mmHg based on resting BP. After 5 min, deflation was automatically recorded for the next 2 min to evaluate the diameter and blood flow rate, and the calculated FMD (FMD = [reactive hyperemia diameter − baseline diameter] × 100%) was used. Resting FMD was measured after resting for at least 30 min, and after-exercise FMD was measured within 10 min of the completion of exercise for each trial. The formula used was %FMD = (Maximal diameter [mm] − Resting diameter [mm]/Resting diameter [mm]) × 100 [40].

### 2.4. Statistical Analysis

All statistical analyses were conducted using SPSS version 28.0 (IBM Corp., Armonk, NY, USA). Data are presented as mean ± standard deviation. The normality of the distribution of all outcome variables was verified using the Shapiro–Wilk W-test before the parametric tests. First, a paired *t*-test was used to compare the mean exercise load during the PLA trial with that of the NRBRJ trial. Second, a two-way analysis (supplement × time) of variance (ANOVA) with repeated measures was used to assess the presence of interactions (supplement × time) and main effects (supplement or time). When the ANOVA revealed a significant interaction or main effect within the supplement, a Bonferroni post hoc test and paired *t*-test were used to identify within-supplement differences at each time point. The effect size in ANOVA can be represented as partial eta-squared values, and the effect size was computed as partial eta-squared values (*η_p_*^2^; small: ≥ 0.01, medium: ≥0.06, large: ≥0.14). Partial eta-squared is a measure of the proportion of the total variance in a dependent variable that is associated with the effect of an independent variable after accounting for other variables [41]. The level of significance was set a priori at *p* < 0.05.

## 3. Results

### 3.1. Mean Exercise Load during Submaximal Exercise

**Figure 3:**
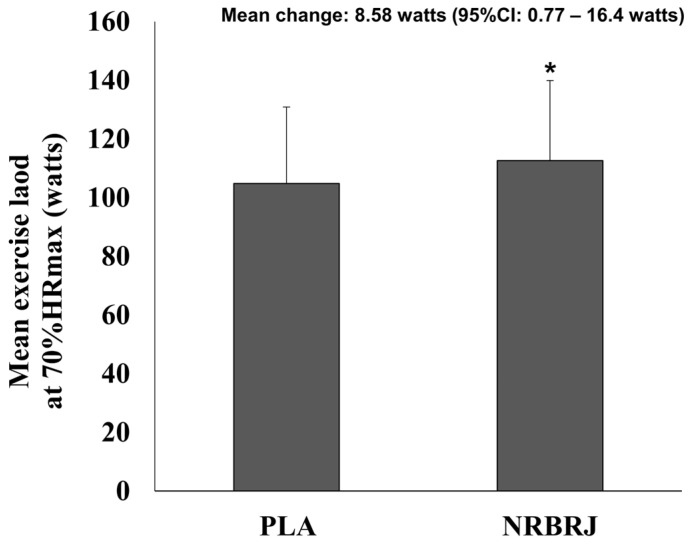
Mean exercise load at 70% maximal heart rate during the 30 min exercise between two trials. CI: confidence interval; NRBRJ: nitrate-rich beetroot juice; PLA: placebo; HRmax: maximal heart rate;. * Significant difference between the PLA and NRBRJ supplement, * *p* < 0.05.

A significant difference was observed in the mean exercise load at 70% HRmax during the 30 min exercise between the two trials (Figure 3). The NRBRJ supplement demonstrated a greater increase in exercise load (mean change, 8.58 watts; 95% confidence interval, 0.77, 16.4; *p* < 0.05) than the PLA supplement.

### 3.2. Cardiovascular Function before, during, and after Submaximal Exercise

#### 3.2.1. Cardiovascular Function during Exercise

**Figure 4:**
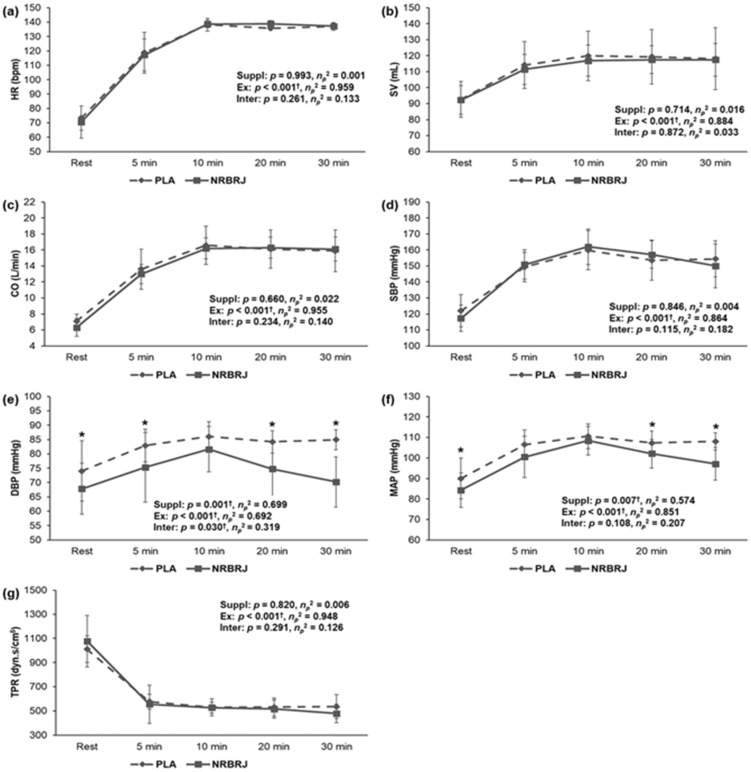
Cardiovascular function during exercise at 70% maximal heart rate between two trials. (**a**) Change in HR during exercise in each trial. (**b**) Change in SV during exercise in each trial. (**c**) Change in CO during exercise in each trial. (**d**) Change in SBP during exercise in each trial. (**e**) Change in DBP during exercise in each trial. (**f**) Change in MAP during exercise in each trial. (**g**) Change in TPR during exercise in each trial. NRBRJ: nitrate-rich beetroot juice; PLA: placebo; HR: heart rate; SV: stroke volume; CO: cardiac output; SBP: systolic blood pressure; DBP: diastolic blood pressure; MAP: mean arterial pressure; TPR: total peripheral resistance; Suppl: supplementation; Ex: exercise; Inter: interaction. ^†^ Significant interaction or main effect of supplement or exercise, ^†^ *p* < 0.05. * Significant difference between the placebo and NRBRJ supplement, * *p* < 0.05.

Figure 4 shows a significant interaction in DBP (*p* = 0.03, *η_p_*^2^ = 0.319) and a significant main effect within each supplement in DBP (*p* = 0.001, *η_p_*^2^ = 0.699) and MAP (*p* = 0.007, *η_p_*^2^ = 0.574). In the post hoc analysis, the NRBRJ supplement significantly decreased DBP (*p* < 0.05) at rest and at 5, 20, and 30 min of exercise compared with the PLA supplement. Additionally, the nitrate supplement showed a significantly lower MAP (*p* < 0.05) than the PLA supplement at rest and after 20 and 30 min of exercise. However, no significant difference was found in HR, SV, CO, SBP, or TPR during the 30 min exercise at 70% HRmax between the PLA- and NRBRJ-supplemented groups.

#### 3.2.2. Pulse Wave Velocity and Flow-Mediated Dilation before and after Exercise

**Figure 5:**
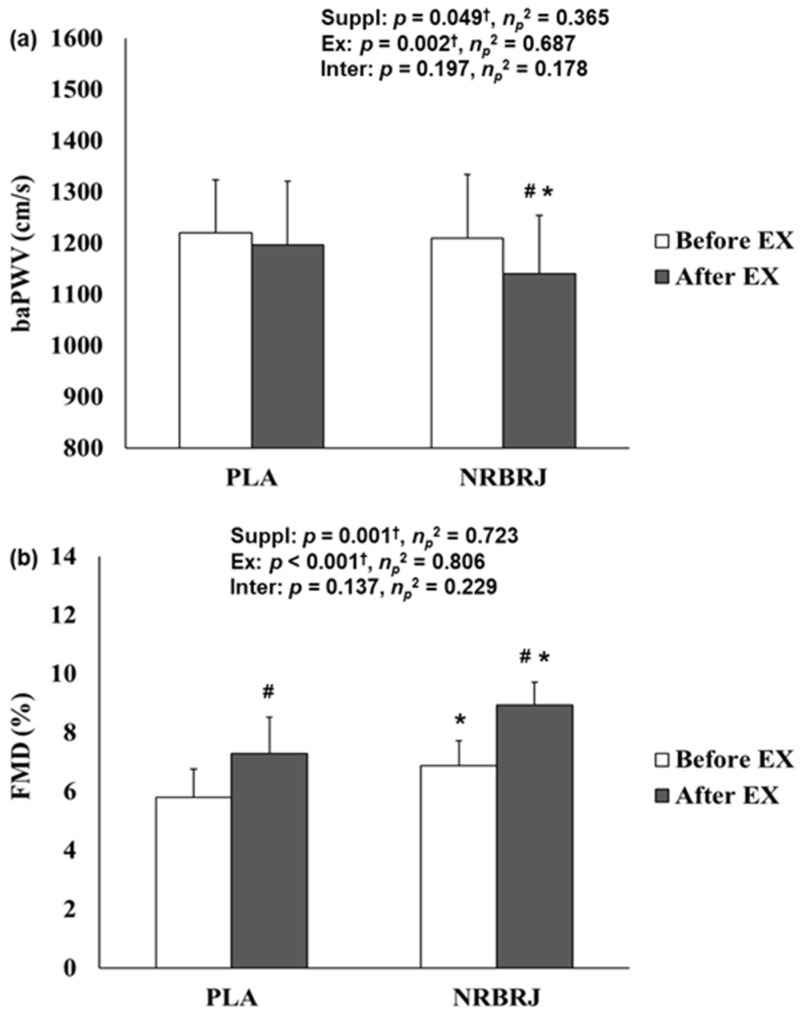
The baPWV and FMD before and after exercise between two trials. (**a**) Change in baPWV in each trial. (**b**) Change in FMD in each trial. NRBRJ: nitrate-rich beetroot juice; PLA: placebo; baPWV: brachial–ankle pulse wave velocity; FMD: flow-mediated dilation; Suppl: supplementation; Ex: exercise; Inter: interaction. ^†^ Significant interaction or main effect of supplement or exercise, ^†^ *p* < 0.05. # Significant difference between before and after exercise in each trial, # *p* < 0.05. * Significant difference between the placebo and NRBRJ supplement, * *p* < 0.05.

No significant interaction was noted between supplementation and exercise in terms of baPWV or FMD (Figure 5). However, a significant main effect within each supplement was found in baPWV (*p* = 0.049, *η_p_*^2^ = 0.365) and FMD (*p* = 0.001, *η_p_*^2^ = 0.723). Post hoc analyses showed that baPWV significantly decreased in the NRBRJ trial (*p* < 0.05) and that baPWV showed a significantly lower value after exercise in the NRBRJ supplementation than in the PLA supplementation (*p* < 0.05). FMD significantly increased via the PLA (*p* < 0.05) and NRBRJ trials (*p* < 0.05); however, NRBRJ supplementation demonstrated a significantly higher FMD value before (*p* < 0.05) and after exercise (*p* < 0.05) than PLA supplementation.

## 4. Discussion

According to our study hypothesis, NRBRJ supplementation and exercise may induce additive improvements in cardiovascular function parameters in healthy men compared to PLA supplementation and exercise. Consistent with this hypothesis, the findings of this study revealed that acute dietary $NO_{3}^{-}$ supplementation and exercise increased the mean exercise load and decreased DBP and MAP during 30 min of submaximal endurance exercise at 70% HRmax compared with PLA supplementation. Additionally, the NRBRJ supplementation demonstrated greater improvements in baPWV and FMD than the PLA supplementation.

Compared to the PLA supplementation, the $NO_{3}^{-}$ supplementation achieved a higher mean exercise load during submaximal endurance exercise with 70% HRmax, which aligns with previous findings [42]. In the preceding study, the NRBRJ supplement group conducted a 30 min cycling session at Wmax 45% and Wmax 65%, followed by a 10 km time trial (TT). The results showed that the NRBRJ supplementation group improved power output and TT performance while decreasing VO_2_ during submaximal exercise [42]. In another study, NRBRJ supplements improved the performance in a 50-mile cycling time trial, achieving a higher average exercise load during submaximal endurance exercise [43]. In this study, VO_2_ was not measured; hence, confirming whether there was a decrease in VO_2_ was not possible. However, the fact that the NRBRJ supplementation group attained a higher average exercise load during submaximal endurance exercise aligns with previous research.

NRBRJ supplementation is believed to enhance higher exercise economy, resulting in a greater power output at the same oxygen consumption or HR levels. This is considered a crucial factor influencing cardiovascular endurance [44]. Moreover, NRBRJ supplementation can potentially convert into a potent vasodilator [45], NO, increasing blood flow to muscle tissues. This aspect allows for a more efficient removal of waste products from muscles through enhanced blood circulation, delaying the onset of fatigue [29]. Cermak et al. [42] suggested that NO can reduce the “slippage” of mitochondrial proton pumps or weaken the expression of uncoupling proteins, thereby lowering the total ATP cost of muscle-force production and improving the efficiency of oxidative phosphorylation. Another study reported that it can improve exercise performance by improving skeletal muscle mitochondrial efficiency [42,46,47]. Considering these factors, in this study, it can elucidate the observed higher power output during cycling in the NRBRJ supplementation group.

Previous studies on the effects of NRBRJ supplementation on cardiovascular parameters have yielded interesting results. In this study, NRBRJ supplementation considerably lowered DBP at rest and during various exercise intervals, which is consistent with previous findings [36,48]. The $NO_{3}^{-}$ consumed through NRBRJ supplementation can generate exogenous NO via the $NO_{3}^{-}$–$NO_{2}^{-}$–NO pathway. The produced NO has the potential to enhance endothelial cell function, reducing peripheral resistance, which is a crucial factor in regulating DBP [49]. Furthermore, after being absorbed into the bloodstream, it can be taken up by vascular smooth muscle cells, activating the NO-cGMP-protein kinase G signaling pathway. This activation can induce vasodilation, potentially influencing blood pressure [50].

In this study, a decrease in SBP was not observed; however, the effects of a reduction in both SBP and DBP after NRBRJ consumption have been well established through numerous studies [11,51]. Most studies involved measuring blood pressure after consuming NRBRJ at rest, and, in a previous study that measured blood pressure during exercise, no changes were noted in either SBP or DBP [52]. These results were similar to a previous study that investigated blood pressure responses to HR during exercise in a population with normal blood pressure (men: 1033 participants) [52,53]. Moreover, the $NO_{3}^{-}$ quantity contained in NRBRJ may also be associated with the observed effects. However, in this study, the rationale for the lack of decrease in SBP remains limited. Additionally, this study found that NRBRJ supplementation did not increase CO, which is contrary to the previous study by Zamani et al. [54] that reported $NO_{3}^{-}\;$supplementation increased CO. This discrepancy in results may be due to the differences in exercise intensity. In the moderate-intensity exercise (70% HRmax) used in this study, Type Ⅰ muscle fibers, which use oxygen efficiently, are primarily activated, potentially limiting the additional effects of $NO_{3}^{-}$ supplementation [55]. In contrast, high-intensity exercise activates Type Ⅱ muscle fibers, which have high oxygen and energy demands, allowing nitrate supplementation to significantly enhance performance [56]. Furthermore, the results of this study showed improvements in FMD following NRBRJ supplementation and exercise. Improvements in FMD suggest an increase in blood flow from arterioles to capillaries and from capillaries to muscle fibers [57,58].

Regarding vascular function and compliance, NRBRJ supplementation resulted in a decrease in baPWV after exercise compared to PLA supplementation. Limited research has been conducted on the effects of $NO_{3}^{-}$ supplementation on PWV before and after exercise. A decrease in PWV implies enhanced vascular compliance, which is influenced by various factors. Some studies have indicated that PWV reduction may be due to changes in the aortic distending pressure [59]. The consumed $NO_{3}^{-}$ may also reduce PWV by regulating sympathetic nervous system tension [60]. Furthermore, an increase in FMD, an indicator of endothelial function, was observed in this study during both rest and after-exercise periods with NRBRJ supplementation. The NO generated after $NO_{3}^{-}$ intake can regulate the expression of vascular endothelial growth factor (VEGF) and inhibit ROS production [61,62]. Elevated ROS levels impair endothelial function, whereas NO can activate antioxidant defense systems, thereby reducing the ROS production rate and promoting an increase in FMD [63,64].

In this study, we explored the effects of NRBRJ supplementation on cardiovascular function before, after, and during exercise. The research findings showed that NRBRJ supplementation, compared to PLA supplementation, led to a greater power output during exercise. Additionally, it significantly reduced DBP during both rest and exercise and was found to decrease baPWV velocity through enhanced vascular compliance. These results indicate that NRBRJ supplementation may enhance beneficial effects on cardiovascular function, thereby increasing aerobic capacity during exercise. The results of this study can provide useful reference material for the general public and athletes when selecting sports supplements.

## 5. Limitations

The present study has several limitations. (1) In this study, we asked participants to limit their intake of products such as broccoli and cauliflower that interfere with the reduction in $NO_{3}^{-}$-$NO_{2}^{-}$-NO. Additionally, the use of mouthwash was restricted during study participation [16]. However, we did not equally restrict the diet of the participants over the procedure of the study. (2) Since plasma and urine measurements were not conducted in this study, we were unable to determine the changes in $NO_{3}^{-}$- and $NO_{2}^{-}$ concentrations in plasma and urine following NRBRJ supplementation. (3) The normality of the distribution of all outcome parameters was verified using the Shapiro–Wilk W-test prior to the parametric tests; however, the small sample size may limit the interpretation of the results of this study.

## 6. Conclusions

This study confirmed that NRBRJ supplementation is effective in improving aerobic exercise capacity and cardiovascular function in healthy males. However, to better understand the combined effects of NRBRJ supplementation and exercise, further research is warranted on the effects on exercise capacity and cardiovascular function considering different factors (e.g., age, sex, environment, and aerobic exercise capacity of participants).

## Figures and Tables

**Table 1:** Participant characteristics (mean ± standard deviation).

Parameters	Healthy Men (n = 12)	Minimum	Maximum
Age (year)	25.1 ± 2.3	21.0	29.0
Height (cm)	179.9 ± 4.4	172.1	185.2
Weight (kg)	83.6 ± 13.3	69.2	110.6
BMI (kg/m^2^)	25.8 ± 3.6	21.1	32.6
Fat free mass (kg)	67.7 ± 6.1	57.3	77.1
Body fat mass (kg)	16.0 ± 9.2	6.4	33.5
Percent body fat (%)	18.2 ± 7.5	7.7	30.3
70% HRmax (bpm)	136.5 ± 1.5	134.0	139.0

Note. BMI = body mass index, HRmax = maximal heart rate.

## Data Availability

The data presented in this study are available upon request from the first or corresponding author.
